# What Are the Criteria for an Acute Form of Anterior Cruciate Ligament Tear for the Severity of the Process by Gait Analysis Data?

**DOI:** 10.3390/jcm12144803

**Published:** 2023-07-20

**Authors:** Dmitry Skvortsov, Alyona Altukhova, Sergey Kaurkin, Alexander Akhpashev

**Affiliations:** 1 Federal Research and Clinical Centre of Russia’s Federal Medical-Biological Agency (FNKC FMBA), Orekhoviy Bulvar 28, Moscow 115682, Russia; 2 Rehabilitation Department, Pirogov Russian National Research Medical University RNRMU, Ul. Ostrovitianova 1, Moscow 117997, Russia; 3 Department of Traumatology and Orthopedics, Academy of Postgraduate Education at FNKC FMBA RF, Moscow 117198, Russia

**Keywords:** knee joint, anterior cruciate ligament tear, acute form, gait analysis

## Abstract

Purpose: There is still controversy over the criteria for acute ACL tear. In this paper, knee joint function and walking were considered possible ones. Method: The study included 21 subjects with acute ACL tear and 20 healthy volunteers as a control group. Biomechanical gait analysis was performed using the inertial sensor system including EMG recording. All subjects (but for controls) were divided into two groups: Group 1—“up to 4 weeks” and Group 2—“from 4 weeks to 3 months”. Results: Temporal gait parameters in subjects from Group 1 demonstrate the asymmetry of 4% and more in terms of the gait cycle with a decrease in the affected limb, and are within normal range in Group 2. The amplitudes at the hip and knee joints in the affected limb are reduced which is especially pronounced in Group 1 (2–4 and 6–10 degrees, respectively). The affected knee joint shows a decrease in the range of motion by up to 5 degrees in the first half of the stance phase and flexion by less than 40 degrees in the swing phase. The tibialis anterior and quadriceps femoris muscle function is decreased in the affected limb only in Group 1 (72% and 78% from normal, respectively). Conclusions: The severity of the condition after an ACL tear is largely determined by functional changes. The time factor is of secondary importance.

## 1. Introduction

The number of knee joint (KJ) injuries with anterior cruciate ligament (ACL) damage has grown in recent years and makes up approximately 50% of all KJ injuries, being significantly higher among women [1,2].

In terms of the main classifications, ACL injuries are distinguished according to the time elapsed from the moment of injury. According to the literature at present, there are no precise criteria for the time-based classification of ACL injuries. Thus, Dae-Hee Lee et al. [3] indicated that ACL tears are considered acute up to three months from the moment of injury, and beyond three months they should be regarded as chronic. Strobel [4] indicated in his manual that the acute phase of ACL tears will be considered for up to three weeks from the moment of injury; following that, it transits to the chronic phase. Cipolla et al. [5] also differentiated between ACL injuries by the time from the moment of injury distinguishing the acute (up to two weeks), subacute (from two to six weeks with the restoration of the full range of motion in the KJ), subchronic (more than six weeks, but without episodes of KJ twisting or instability), and chronic (after a repeated episode of KJ injury/twisting/instability) phases of the injury. Based on the data presented in the paper by Shelbourne et al. [6], an indirect conclusion can be achieved that the acute phase of ACL injury lasts at least three weeks from the moment of injury.

Thus, in the literature, there are different approaches to defining the acute phase of ACL tears. Most authors define it as a period of one to three months after the injury with a minimum of three weeks [4] and a maximum of up to seven months after the injury [7]. At the same time, a significant, and sometimes the only, importance is assigned to the factor of time elapsed since the moment of injury.

Biomechanical gait analysis in the acute phase of ACL tears has received very little coverage in the literature. ACL tears can obviously pose a danger of achieving secondary damage. Some studies lack any information on the time elapsed since the injury by the time the subjects were examined [8]. However, according to the biomechanical data provided, the examined group showed chronic ACL insufficiency when the gait function was mainly restored. Christian J et al. analyzed in their study [9] the biomechanical gait parameters in subjects with a recent ACL tear. The mean time from the moment of the tear to an examination made up 21 days. However, this study was devoted to the possibility of the automatic analysis of gait changes in these subjects. For this reason, some particular parameters were not covered by the publication. Gardinier ES et al. in their study [7] analyzed gait biomechanics in subjects with ACL tears within 7–8 weeks after the injury. They found out that in the stance phase, the amplitudes of KJ first flexion and subsequent extension were reduced (in our study, these are the parameters *Ka1* and *Ka2*, respectively). The muscles of the thigh showed a decrease in both maximum flexion and extension during walking. Meanwhile, no isolated decrease in the strength of the flexors or extensors of the knee joint was registered. No significant changes in the kinematics of the KJ during walking were found in average terms about 8 weeks after the ACL tear [10]. The same result was obtained for walking speed and cadence. According to one study [11], at 7.0 ± 11.5 weeks after an ACL tear, the amplitude for KJ motion in the sagittal plane during walking reduced due to a decrease in the amplitude of the first flexion and subsequent extension. Moreover, the joint was initially slightly flexed. A little earlier, another gait investigation was performed [12]. The mean time from injury was 3.4 weeks. The authors noted a decrease in the amplitude of the first flexion *Ka1* and subsequent extension *Ka2* (underextension). Such symptoms of KJ function decrease have been noted by most researchers, not only in acute ACL damage [13] but also in such a common disease, such as KJ osteoarthritis [14]. We also found data on these kinematic changes in our prior studies, both in subjects with acute ACL injuries [15] and KJ osteoarthritis [16]. In the previous study of gait biomechanics in the acute phase of ACL tears [15] (with an average time of 16 days after injury), we assumed that KJ stiffness in this period could be a result only of muscle tension. The KJ muscles keep the joint from moving, leaving a much lower amplitude while walking. This assumption comes into some conflict with the arthrogenic muscle inhibition (AMI) concept [17,18]. The AMI concept assumes KJ muscles’ and, above all, quadriceps’ femoris inhibition. However, it remains unclear what then restricts KJ motion. Lepley AS and Lepley LK [19] provided in their review a description of the complex neural mechanisms of AMI. At the same time, the main KJ stabilizer—the quadriceps femoris muscle—is inhibited, which significantly determines the presentation of AMI. However, this comes into some conflict with the experimental study [20]. In this study, no supraspinal effect on quadricep AMI was found. The EMG activity of muscles in the acute phase of ACL tears also remains insufficiently studied, especially if the acute phase is admitted as a time period of around the first 4 weeks.

Another point disclosed in our previous paper [15] was the time-based aspect. The time from the injury certainly makes a difference. Sooner or later, even without the necessary conservative treatment, pain decreases and there is a gain of motion in the KJ. However, we assume that the time-based factor for determining the severity of the process exactly is not the determining one. We attempt to test this hypothesis.

Study hypothesis. We assume that the severity of the process will be determined based on, among other things, the biomechanical functional parameters of walking and knee joint kinematics. Moreover, we assume that, in the early stage of ACL tears, the function of the muscles of this lower limb is also reduced.

## 2. Materials and Methods

The study was conducted from 2020 to 2023 at the Federal Research and Clinical Centre of Russia’s Federal Medical Biological Agency of Russia.

The study population included 21 subjects (12 males and 9 females) with acute ACL tears at a mean age of 36 years old (range: 18–54 years old).

Inclusion criteria: male and female aged 18 to 55 years old; ACL tears confirmed by MRI for no more than 3 months; and the presence of primary or post-traumatic KJ osteoarthritis grades 0–II by Kellgren–Lawrence grading system.

Exclusion criteria: age under 18 and over 65 years old; other KJ ligament injuries; osteochondral defects of the knee joint; primary or post-traumatic osteoarthritis of one or both KJs grades III–IV by Kellgren–Lawrence grading system; hip or ankle joint disorders; chronic inflammatory diseases of the musculoskeletal system; and spine diseases leading to a significant violation of the independent movement of the subject.

The healthy control group included 20 healthy subjects (10 females and 10 males), with a mean age of 28.8 ± 3.6 years old (23–35 years old) without a past history of injuries or musculoskeletal diseases.

The study was conducted according to the principles of the Declaration of Helsinki and was approved by the local ethics committee. All subjects signed an informed consent before the tests.

The examined subjects with verified ACL tears presented with heterogeneous clinical symptoms. Some used orthoses and additional walking aids for two or three weeks and had significant KJ edemas, as well as experienced significant pain. Others could walk with a full axial load on the affected lower limb, but showed some restrictions in the range of motions and experienced moderate pain. Yet, others complained of slight pain, had no edema, and could walk without any restrictions almost on the second or third day after the injury.

Of the 21 subjects surveyed, 2 were athletes, 1 was engaged in physical labor, while the remaining 18 worked in an office and practiced physical activities at a non-professional level.

All subjects from the study group were able to impose the axial load on the affected limb within the limits of activities of daily living. At the same time, all subjects, except for one, experienced pain of varying severity in the period after the injury and at the time of the examination. Seven subjects from the study group noted a limitation of knee joint range of motion, and only one subject resorted to the use of an orthosis and crutches in the period before the consultation.

There was one subject with grade-I and one with grade-II arthritis in the study group.

The healthy control group included 20 healthy subjects (10 females and 10 males) with a mean age of 28.8 ± 3.66 years old (23–35 years old), of a mean height of 176.8 ± 5.53 cm (168–188 cm), with a mean weight of 76.25 ± 14.09 kg (55–100 kg), with no past history of injuries or musculoskeletal diseases.

All examined subjects were divided into two groups according to the time elapsed after injury: group 1—“up to 4 weeks” (11 subjects)—and group 2—“from 4 weeks to 3 months” (10 subjects). As per Dae-Hee Lee et al. [3], 3 months after, the injury was designated as a cutoff. Based on the literature and our own experience, 4 weeks from the moment of injury was proposed to define the most acute phase. This was a kind of compromise between the various points of view outlined above.

In the group of subjects examined during the period of up to 4 weeks from the date of injury (group “up to 4 weeks”), 10 subjects had an indirect mechanism of the KJ injury, and 1—a direct mechanism of injury. The age of the subjects ranged from 21 to 54 years old with a mean of 34.5 years. The group included 7 men and 4 women. Their height ranged from 160 to 190 cm with a mean of 175 cm. Their body weight ranged from 53 to 129 kg with a mean of 76.0 kg. Nine subjects had an ACL tear in the left KJ, while two had that in the right one.

In the group of subjects examined during the period of 4 weeks to 3 months from the injury (group “from 4 weeks to 3 months”), 9 had an indirect mechanism of knee joint injury. The age of the subjects ranged from 18 to 55 years old with a mean of 38.9 years. The group included 5 women and 5 men. Their heights ranged from 162 to 180 cm with a mean of 168.8 cm. Their body weight ranged from 57 to 108 kg with a mean of 74.8 kg. Nine subjects had a damaged right knee joint and one—the left one. In this group, three subjects did not experience a feeling of instability in the joint. Nine subjects complained of pain.

The clinical study was carried out in a standard manner. A visual analog scale (VAS) [21] and IKDC 2000 scale (IKDC knee examination form) [22] for objectivation, as well as a single-leg vertical hop test (SLVHT) [23,24], were used. For a subsequent analysis, the following parameters were recorded for each subject: the period from the moment of injury to the initial appointment in days and months for a group of subjects with that of “up to 4 weeks” and “from 4 weeks to 3 months”, respectively; the presence or absence of edemas at the time of the examination; the ability to impose a full (body weight) axial load on the limb; passive extension insufficiency; passive flexion insufficiency; use of additional walking aids; assessment of pain at the time of injury using the VAS scale; assessment of pain at the time of the examination using the VAS scale; and single-leg vertical hop test.

**Figure 1:**
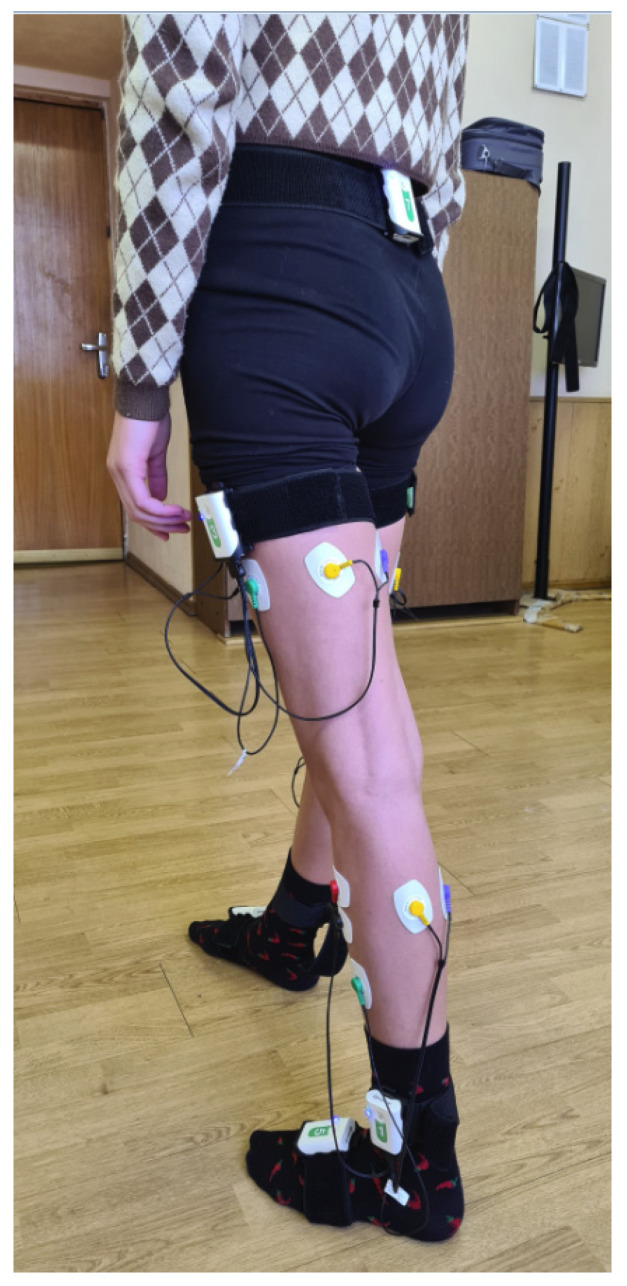
The subject during the examination as per the method with registration in the hip and knee joints, and EMG of the main flexor–extensor muscles.

A biomechanical gait analysis was performed using the Stadis system (Neurosoft, Ivanovo, Russia). Neurosens inertial sensors were attached to the subject’s sacrum, the outer middle-third of the thigh, the outer ankle, and the foot instep, on both sides (Figure 1). A total of seven sensors were used. Each sensor also had two channels for EMG data. The thigh-located sensors captured the EMG signals of the rectus femoris and joint activity of the hamstring muscles. The lower-leg-located sensors captured the EMG signals of the anterior tibial muscle, as well as the joint activity of the external and internal heads of the triceps surae. Disposable Medico electrodes were used for EMG recording. We analyzed the maximum amplitude of each muscle (μV) based on smoothed rectified EMG normalized to the gait cycle and did the same with the goniograms.

The upright standing position with hips and knees straightened was assumed as neutral (calibration position). Then, biomechanical gait parameters were recorded. The subjects were instructed to repeatedly walk a distance of 10 m at an arbitrary pace, turning around at the end of the distance and continuing to walk. Even though all the subjects arrived to the clinic on their own feet, they were first asked if they would be able to perform this test. Of the 21 subjects, only 1 used a cane. None of the subjects were re-injured or developed any other additional disorders during the gait study.

Steps with unsteady parameters (acceleration or deceleration) were automatically excluded from the analysis. The remaining walking cycles were calculated. On average, the recording was completed upon reaching at least 30 walking cycles. The software was based on a verified neural network algorithm for the gait cycle (GC) analysis. It calculated the GC of each leg and other GC parameters. The following biomechanical parameters were selected for the subsequent analysis: GC duration, s; individual GC phases and periods were measured as percent of GC: stance phase (SP), single support (SS), and double support (DS); and walking speed (V), km/h. Kinematic parameters were captured for the hip, knee, and ankle joints in the sagittal plane (flexion–extension), the joint goniogram was plotted over a gait cycle, and the following parameters were calculated automatically:

– The hip joint: maximum amplitude over GC (HA, degrees) and the phase of maximum hip extension (HP).
– The knee joint: first flexion amplitude (*Ka1*) and its phase (*Kp1*), extension amplitude (*Ka2*) and its phase (*Kp2*), and swing flexion amplitude (*Ka3*) and its phase (*Kp3*).
– The ankle joint: amplitude (AA) over GC.

The envelope EMG was analyzed for maximum amplitudes developed over GC, μV, by the tibialis anterior muscle (TA), calf muscles, gastrocnemius muscles (GAs), quadricep femoris muscles (QAs), and hamstring muscles (HMs).

The obtained data were analyzed by ANOVA using the Statistica 12 software. The medians and quartiles (the 25th and 75th percentiles) were calculated. Due to the small sample size, the normality of the data was not assessed; thus, the significance of the differences was assessed using the Wilcoxon–Mann–Whitney test with a *p*-value < 0.05 considered significant. A comparative assessment of similar parameters of affected and intact limbs in the groups “up to 4 weeks” and “from 4 weeks to 3 months”, of the affected and intact sides within each group, and of similar parameters of the affected and intact sides of both groups with those of the control group was conducted.

## 3. Results

### 3.1. Results for Both Groups

The clinical status of the examined subjects is presented in Table 1.

According to the data presented in Table 1, most of the examined subjects neither had joint edemas nor showed axial load limitations at the time of the examination. The amplitude of the passive extension was also impaired in isolated cases. At the same time, the impairment of passive flexion amplitude showed high variability, up to severe limitations (D) in the group “up to 4 weeks”. In the group “from 4 weeks to 3 months”, only one subject showed mild limitations (B). The VAS scale showed no significant patterns. In the group “from 4 weeks to 3 months”, there were more subjects able to perform the single-leg vertical hop test.

The results of the biomechanical analysis are presented in Table 2, Table 3 and Table 4.

When comparing the two groups, the GC values in both groups showed no significant changes, but were symmetrically increased in both groups when compared with the control group. SP was significantly shorter in the affected limb in the group “up to 4 weeks” compared to that in the group “from 4 weeks to 3 months”. Moreover, in the group “up to 4 weeks”, SP was significantly shorter in the affected limb compared to that in the intact one for the subjects from the same group, which indicated the presence of asymmetry. The SS parameter also showed asymmetry in the form of significant shortening in the affected limb compared to the intact one in the group “up to 4 weeks”. The single support phase (SS) was significantly longer in the intact limb in the group “up to 4 weeks” compared to that in the group “from 4 weeks to 3 months”. There were no significant changes in the DS index in both study groups; however, at the same time, it was symmetrically increased in both groups when compared to the control group. Walking speed was significantly lower in both groups when compared to the control group.

The data on the range of motion in the hip, knee, and ankle joints are presented in Table 3.

There were no significant differences between two groups in the range of motion in hip and ankle joints. There were only significant differences from the control group and the intact limb.

In the group “up to 4 weeks”, the *Ka1* amplitude in knee joints was significantly lower in the affected limb compared to the intact one (asymmetry). The phase of this amplitude was significantly reduced in the affected limbs compared to the control group. *Ka3* amplitude and *Kp3* phase were significantly lower on the affected side in the group “up to 4 weeks” compared with those in the group “from 4 weeks to 3 months”.

The results of the muscle EMG activity analysis are presented in Table 4.

In the group “up to 4 weeks”, the amplitude of the tibialis anterior muscle was significantly lower compared to that in the control group, and the amplitude of the quadriceps muscle was significantly reduced in the injured limb compared to that in the intact one (asymmetry). In the group “from 4 weeks to 3 months”, no significant differences in the values of EMG activity were noticed when compared to both the control group and the intact limb.

### 3.2. Examples of Typical Clinical Cases

An example of individual variability was demonstrated by hip and knee joint functions in four subjects.

**Figure 2:**
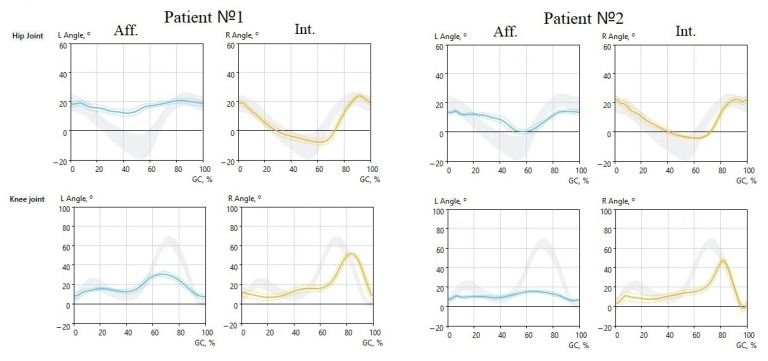
Goniograms of the hip and knee joints of subjects 1 and 2 (the left limb is affected). Blue line—left side, yellow—right side. The vertical scale is in degrees; the horizontal scale is % of GC. Subject 1–4 days after injury; used crutches. Subject 2–6 days after injury.

**Figure 3:**
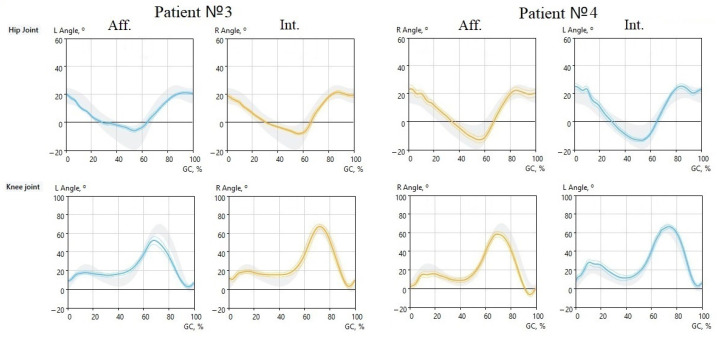
Goniograms of the hip and knee joints of subjects 3 and 4. Blue line—left side, yellow—right side. Aff., affected limb; Int., intact limb; vertical scale in degrees; horizontal scale in % of GC.

Goniograms of the hip and knee joints show developing changes (Figure 2 and Figure 3).

Subject 1 walked with the help of crutches. Therefore, the hip joint amplitude in the affected limb was insignificant, as well as the motion in the slightly bent KJ. The intact limb showed a normal range of motion in the hip joint. Subject 2 showed similar changes with the difference that he no longer used crutches; however, the motion in both hip and joints in the affected limb were significantly reduced.

While subjects 1 and 2 showed a typical pattern of impaired gait function, subjects 3 and 4 were examined just two days after the injury. Nevertheless, their performances quite slightly differed from the control group (Figure 3).

Both subjects had acute ACL tears. Subject 3 demonstrated obvious symptoms of the injury, such as the slight initial flexion of the left KJ (symmetrical), decreased amplitudes of both flexions, and almost no extension. Subject 4 demonstrated almost no decrease in the function of the affected right KJ. Only a slight decrease in the first and second bending amplitudes could be noted, which reached the lower limit of the control group.

## 4. Discussion

The results obtained in the present study are similar to those in [7] in terms of a decrease in the first two amplitudes in the affected, as well as in the function of, the flexor–extensor muscles. However, these data were obtained over a period of 7–8 weeks after the injury. In the present study, only the group “up to 4 weeks” showed such results, while another group, similar to that in [7] in terms of the time period, showed no significant decrease in muscle activity compared to the intact limb or the control group. This significantly agrees with the results of the study [10], where no significant changes were noted in the biomechanics of walking in subjects with a period of 8 weeks after the ACL tear. However, in the present study, minor symptoms persisted during these periods. A decrease in the values of the first two KJ amplitudes in the early period after injury was also noted in [7,15]. We assumed that it was the flexion–extension amplitudes that were the first to respond to joint pathology, both acute and chronic, such as arthritis [16,25]. A decrease in the basic amplitudes was the most universal joint response to injury.

From the clinical perspective, subjects from the group “from 4 weeks to 3 months” from the moment of injury had milder symptoms and showed better functionality; although, the differences were not strongly marked. At the same time, the restriction of passive flexion in an earlier period was more often limited in the group “up to 4 weeks”, as was the ability to perform the SLVHT test on the affected leg. However, even in this case, very high variability was noted.

In the group “up to 4 weeks”, the motion of the hip and knee joints was significantly more limited in the affected limb than that in the intact one. However, it was significantly greater in the ankle joint, which can be attributed to compensation for the missing flexion in the affected knee joint. In the group “up to 3 months”, the range of motion in all three joints was symmetrical. There were no significant differences anymore. There were some insignificant differences compared to the control group, which alone distinguished this group from healthy people.

From a functional point of view, an acute process (the group “up to 4 weeks”) was characterized by an increase in the duration of the GC from a value of 1.3 seconds, asymmetry of the SS with its shortening in the affected limp, and its increase in the intact one. According to the present study, this asymmetry can range from 4% of the GC and higher. A similar asymmetry was also noticed for SS and DS phases. Despite the fact that the walking speed was significantly reduced in both groups, it showed significant variability and could only be used indirectly. The range of motion in hip joints was asymmetric with a decrease in the affected limb; however, the asymmetry value of 2 to 4 degrees could be taken as an indirect symptom. The same can be achieved with the amplitudes of the first flexion and subsequent extension of the knee joint. Control variation in this case can vary in the range from 6 to 10 degrees. However, the difference between these amplitudes is more significant. In fact, there is a reduction in extension; thus, the amplitude difference in the affected KJ is in the range from 0 to 5 degrees with the total norm at a rate of 10. The most significant is a decrease in the amplitude of KJ flexion during the swing phase. This amplitude can be no greater than 40 degrees and show asymmetry compared to an intact limb greater than 5 degrees. At the same time, the function of the ankle joint in the affected limb showed an increase in amplitude compared to that in the intact one. The asymmetry was not great and could be considered as an indirect symptom. Similar indirect symptoms and decreased activity were registered in the tibialis anterior and quadriceps femoris muscles. Our data to some extent agree with those presented in the study by Gardiner ES et al. [7]. However, the time from injury in the subjects included in their study was long; thus, these subjects can be somewhat considered similar to the subjects from the group “up to 3 months” in the present study. Though, as opposed to Gardiner ES et al. [7], in the group “up to 3 months”, no significant differences in muscle function were noted, both in comparison with the unaffected limb as well as with the controls. At the same time, in the paper by Harato K et al. [11], a decrease in the first two amplitudes in the affected KJ was also noted in later periods.

In terms of up to 4 weeks, there were significant differences in the gait function and kinematics of motion in the joints, characterized by the fact that the function of the entire affected limb decreased. Walking was possible only at a slow pace, there were symptoms of the unloading of the affected side, a decrease, and asymmetry in joint function. A significant part of these symptoms was resolved at a later date. However, this was true only in the present study with the subjects enrolled having practically no other concomitant damage to the structures of the knee joint. In the study by Harato K et al. [11], a significant part of the examined patients had a concomitant meniscal injury, which, according to this work, worsened the function of the damaged knee joint. Thus, they reported a decrease in the first two motion ranges and a more prominent flexion in the initial position of the KJ more than 10 months after the injury.

The given typical clinical cases clearly demonstrated what was concealed by the statistics. In the early stages, these were significant asymmetries of all parameters, aimed at reducing the load on the affected limb. At the same time, some cases appeared paradoxical, since, in some subjects, there were practically no functional symptoms specific to ACL tears in the early stages.

During the first 4 weeks, the function of the joint and the entire lower limb recovered. The subsequent period, in contrast to the previous one, was characterized by relatively insignificant dynamics. If there was no concomitant pathology, complications, or inadequate rehabilitation, then, during the first 4 weeks, there was a recovery close to normal biomechanical parameters, although not reaching them. Both present and previous studies prove that, even in longer periods of 7 or more weeks [11], functional symptoms, which can be assessed by methods of biomechanical gait analysis, remain. However, their sensitivity is very variable. The basic temporal and spatial characteristics of gait are the general parameters that cannot be sensitive to small nuances [10], which can be seen at an early date in our study. The kinematic parameters—the data obtained from the force platform and the developed moments of the joint forces—were more sensible [9,10]. Thus, whether we find any symptoms specific to ACL tears or not, depends on the sensibility of the research method used.

The EMG activity of the affected lower-limb muscles was reduced only in the group “up to 4 weeks” and only in two muscles, i.e., anterior tibial and quadriceps femoris. At the same time, the quadriceps femoris showed values close to the basic tonic activity. This confirmed the AMI (arthrogenic muscle inhibition) concept, according to which this muscle was the most affected [17,18]. The group examined at the later date no longer showed any changes in activity compared to the control group. At the same time, Aghdam HA et al. reported in their study [26] a decrease in the activity of several thigh muscles in the affected limb at a later (up to 6 months) time after the injury.

If all the analyzed parameters are considered, then the boundary of the early period of 4 weeks, set in terms of the present study, is close to defining it as acute. Defining an acute phase up to 7 months after an injury can hardly be justified [7]. It is during this period when consequences can be observed, which can significantly vary from the absence of complaints to the presence of several limitations in the joint function. This study clearly showed that subjects diagnosed with acute ACL tears represented a functionally very heterogeneous group, especially in the first days and weeks after the injury. Moreover, according to the studies by other authors, several functional changes typical of an acute rupture, detected using a biomechanical study of gait, could also be present after 4 weeks. Thus, a very diverse functional picture for a long period after injury can be observed.

The study showed that the gait function and KJ kinematics in subjects with acute ACL injuries are extremely variable. Functional boundaries range from almost complete KJ blockade—with only swinging, forced (under the weight of the body), very limited motion remaining—to almost normal indicators. All the observed variations fit into a very limited time frame.

The limitations of this study are related to the relatively small number of patients examined who were examined once at the time of their admission. At the same time, for all the subjects examined, this examination was not the first one performed immediately after the injury. Another significant limitation was that, under the existing conditions, it was impossible to organize follow-up examinations of subjects during the first weeks or months after injury. We also lacked the technical ability to register a number of important parameters and moments of forces in the joints, as well as analyze the COG and COP data.

The development of precise functional criteria for acute staging requires a significantly larger number of subjects to be analyzed. For example, in our previous work on this topic [15], a different principle of group formation was applied, i.e., based on the maximum amplitude in the KJ. Of the 18 patients examined, in 6, this amplitude did not exceed 20 degrees. In further analyses, subjects in both groups will be made to walk at a low speed, which will affect other parameters.

## 5. Conclusions

Acute ACL injuries in the period of up to 4 weeks are characterized by a functional decrease in walking speed and the presence of characteristic asymmetries with a decrease in parameter values mainly in the affected limb. When it comes to joints, the affected limb is characterized by reduced amplitudes in the hip and knee joints and increased amplitudes in the ankle joint. The function of the tibialis anterior and quadriceps femoris muscles also decreases in the affected limb. At a later date (up to 3 months), the symmetry of the parameters is restored. However, the recovery period for every individual is highly variable.

We can assume that the acute form of ACL tear in its clinical expression is not only determined by the time elapsed since the injury; the acute or chronic form is determined mainly by the functional restructuring that follows the injury. The terms of functional recovery turned out to be too variable to use the time factor to characterize the acuity of the process after injury.

The definition of acute ACL injury should not be based on the only time factor, all other things being equal, but also on the functional state of both the damaged KJ and the entire limb. The best possible option is to perform a biomechanical analysis.

## Figures and Tables

**Table 1:** Clinical characteristics of subjects at the first visit.

Group	Time to Examination	Edema	Axial Load	Passive Extension Deficiency	Passive Flexion Deficiency	Crutches	VAS * at the Time of Injury	VAS at the Time of Examination	SLVHT **
Up to 4 weeks	6 days	None	Full	A	D	None	5	3–4	0
	6 days	None	Full	A	A	None	–	–	–
	7 days	None	Full	A	D	None	8	1	100
	7 days	Yes	Full	A	D	None	7	4–5	0
	7 days	None	Full	A	D	None	9	1	0
	15 days	None	Full	A	A	None	7	2	0
	22 days	None	Limited	B	D	Yes	0	3	0
	2 days	None	Full	A	A	None	0	0	100
	2 days	None	Full	A	A	None	5	5	0
	15 days	None	Full	A	A	None	8	3	0
	7 days	None	Full	A	C	None	1	0.5	30–40
From 4 weeks to 3 months	1 month	None	Full	B	A	None	10	10	10
	1 month	None	Full	A	A	None	10	8	0
	1.5 months	None	Full	A	A	None	0	0	100
	1.75 months	None	Full	A	A	None	3	1	20
	2 months	None	Full	A	A	None	7	0	30
	1.5 months	None	Full	A	B	None	10	4–5	0
	2.5 months	None	Full	A	A	None	10	3	0
	3 months	None	Full	A	A	None	7	4	0
	1.2 months	None	Full	A	A	None	10	3	30
	3 months	None	Full	C	A	None	8	6	0

* VAS, Visual analog scale; ** SLVHT, single-leg vertical hop test.

**Table 2:** Time-based parameters of the gait cycle. The results of comparing parameters in subjects of the groups “up to 4 weeks” and “from 4 weeks to 3 months”.

Parameter	Up to 4 Weeks		From 4 Weeks to 3 Months		Control
	Affected Limb	Intact Limb	Affected Limb	Intact Limb	
Gait Cycle (s)	1.3 (1.1; 1.5) *p* = 0.247 * p = 0.001 ^#^ *p* = 0.974 ^^^	1.3 (1.1; 1.5) *p* = 0.263 * p = 0.001 ^#^	1.2 (1.1; 1.2) *p* = 0.003 ^#^ *p* = 0.94 ^^^	1.2 (1.1; 1.2) *p* = 0.009 ^#^	1.1 (1; 1.1)
Stance Phase (%)	61.3 (59.9; 62.5) *p* = 0.002 * p = 0.026 ^#^ *p* = 0.001 ^^^	67.6 (64; 69.6) *p* = 0.181 * p = 0.001 ^#^	64.9 (64; 65.8) *p* = 0.001 ^#^ *p* = 0.91 ^^^	64.6 (63.6; 65.8) *p* = 0.001 ^#^	62.9 (61.6; 63.7)
Single Support Phase (%)	32.8 (29.4; 36.2) *p* = 0.218 * p = 0.001 ^#^ *p* = 0.001 ^^^	38.7 (36.8; 40.3) *p* = 0.003 * p = 0.045 ^#^	35.3 (34; 36.2) *p* = 0.002 ^#^ *p* = 0.97 ^^^	34.8 (34.1; 36.5) *p* = 0.002 ^#^	37.4 (36.3; 38.3)
Double Support Phase (%)	29.8 (25.1; 32) *p* = 0.888 * p = 0.026 ^#^ *p* = 0.818 ^^^	29.6 (25.7; 32.2) *p* = 1 * p = 0.011 ^#^	29.6 (28.1; 31.6) *p* = 0.001 ^#^ *p* = 0.85 ^^^	29.4 (28.5; 31.9) *p* = 0.001 ^#^	25.5 (24; 27.7)
Speed (km/h)	3.4 (2.5; 4.4) *p* = 0.418 * p = 0.017 ^#^		3.8 (3.7; 4) *p* = 0.006 ^#^		4.3 (4.1; 4.7)

*p*, a level of significance; *, differences from the similar value in subjects from the group from 4 weeks to 3 months; ^#^, differences from the similar value in subjects from the control group; ^^^, differences from a similar value on the intact side in subjects from the same group.

**Table 3:** Motion in the hip, knee, and ankle joints in subjects from the groups “up to 4 weeks” and “from 4 weeks to 3 months”.

Parameter	Up to 4 Weeks		From 4 Weeks to 3 Months		Control
	Affected Limb	Intact Limb	Affected Limb	Intact Limb	
*T_a_*	29 (23; 33) *p* = 0.113 * p = 0.043 ^#^ *p* = 0.237 ^^^	31 (27; 38) *p* = 0.36 * p = 0.507 ^#^	31 (30; 36.5) *p* = 0.846 ^#^ *p* = 0.345 ^^^	35.5 (32; 39) *p* = 0.308 ^#^	32.5 (30.4; 37)
*Kp1*	9.5 (8; 10.6) *p* = 0.398 * p = 0.01 ^#^ *p* = 0.393 ^^^	10.1 (9; 11.6) *p* = 0.778 * p = 0.099 ^#^	10.1 (9.5; 11) *p* = 0.047 ^#^ *p* = 0.762 ^^^	10.1 (9.5; 12.1) *p* = 0.083 ^#^	11.6 (9.5; 15.3)
*Ka1*	8.3 (3.6; 13.7) *p* = 0.062 * p = 0.001 ^#^ *p* = 0.045 ^^^	14 (8; 14.9) *p* = 0.751 * p = 0.128 ^#^	12.2 (8.7; 15.4) *p* = 0.104 ^#^ *p* = 0.571 ^^^	13.2 (11; 15.5) *p* = 0.286 ^#^	14.4 (12.3; 17.2)
*Kp2*	32.7 (28.6; 39.9) *p* = 0.673 * p = 0.059 ^#^ *p* = 0.158 ^^^	36.7 (34.7; 41.7) *p* = 0.86 * p = 0.655 ^#^	34.7 (32.7; 38.2) *p* = 0.133 ^#^ *p* = 0.174 ^^^	39.7 (34.2; 40.9) *p* = 0.577 ^#^	36.7 (34.2; 40.1)
*Ka2*	6.8 (4.8; 8.8) *p* = 0.751 * p = 0.342 ^#^ *p* = 0.2 ^^^	4.8 (1.5; 7) *p* = 1 * p = 0.706 ^#^	6.7 (4.3; 11.9) *p* = 0.124 ^#^ *p* = 0.186 ^^^	4.2 (2.4; 11.4) *p* = 0.961 ^#^	4.5 (1.7; 8.1)
*Kp3*	68.3 (66.3; 69.8) *p* = 0.009 * p = 0.18 ^#^ *p* = 0.008 ^^^	72.4 (69.3; 76.4) *p* = 0.418 * p = 0.009 ^#^	71.9 (69.3; 73.7) *p* = 0.038 ^#^ *p* = 0.821 ^^^	72.4 (67.8; 73.8) *p* = 0.081 ^#^	69.4 (66.8; 71.4)
*Ka3*	48.9 (40.7; 55.9) *p* = 0.027 * p = 0.004 ^#^ *p* = 0.115 ^^^	54.2 (45.8; 58) *p* = 0.342 * p = 0.287 ^#^	57.5 (54.2; 60) *p* = 0.753 ^#^ *p* = 0.678 ^^^	58.6 (55.2; 63.2) *p* = 0.961 ^#^	57.2 (50.4; 64.3)
*AA*	31 (28.5; 32.5) *p* = 0.453 * p = 0.197 ^#^ *p* = 0.596 ^^^	28 (25.5; 32) *p* = 0.659 * p = 0.067 ^#^	31 (30.3; 35.5) *p* = 0.786 ^#^ *p* = 0.253 ^^^	30 (27.5; 33) *p* = 0.048 ^#^	32.8 (30.4; 37)

*Ta*, amplitude of the hip joint over gait cycle; *Ka1*, knee joint first-flexion amplitude; *Kp1*, knee joint first-flexion phase; *Ka2*, knee joint extension amplitude; *Kp2*, knee joint extension phase; *Ka3*, knee joint swing-flexion amplitude; *Kp3*, knee joint swing-flexion phase; *AA*, amplitude of the ankle joint over gait cycle; *p*, a level of significance; *, differences from the similar values in subjects from the group “from 4 weeks to 3 months”; ^#^, differences from the similar values in subjects from the control group; ^^^, differences from a similar value on the intact side in subjects from the same group.

**Table 4:** Muscle electromyography in subjects from the groups “up to 4 weeks” and “from 4 weeks to 3 months”.

EMG	Up to 4 Weeks		From 4 Weeks to 3 Months		Control
	Affected Limb	Intact Limb	Affected Limb	Intact Limb	
Tibialis anterior muscle	95 (81.5; 142.5) *p* = 0.307 * p = 0.047 ^#^ *p* = 0.157 ^^^	148 (102.5; 234) *p* = 0.713 * p = 0.409 ^#^	118.5 (110.3; 141) *p* = 0.291 ^#^ *p* = 0.791 ^^^	120.5 (108; 206) *p* = 0.981 ^#^	131.5 (109.3; 161.5)
Gastrochemius muscles	96 (35; 156) *p* = 1 * p = 1 ^#^ *p* = 1 ^^^	162 (78; 200.5) *p* = 1 * p = 1 ^#^	128 (87; 163.3) *p* = 0.452 ^#^ *p* = 0.385 ^^^	133.5 (115; 212.8) *p* = 0.081 ^#^	114 (85; 142)
Quadriceps femoris muscles	44 (20.3; 66.3) *p* = 0.104 * p = 0.156 ^#^ *p* = 0.045 ^^^	71.5 (56.5; 126.5) *p* = 0.91 * p = 0.079 ^#^	58.5 (41; 99.5) *p* = 0.452 ^#^ *p* = 0.345 ^^^	73 (56; 92) *p* = 0.099 ^#^	56 (28.3; 80.3)
Hamstring muscles	95.5 (31; 125.3) *p* = 1 * p = 1 ^#^ *p* = 0.701 ^^^	138 (86.8; 262.8) *p* = 1 * p = 1 ^#^	103 (71; 142.8) *p* = 0.065 ^#^ *p* = 1 ^^^	81 (65; 109.3) *p* = 0.645 ^#^	74 (48; 98.8)

*p*, a level of significance; *, differences from the similar values in subjects from the group “from 4 weeks to 3 months”; ^#^, differences from the similar values in subjects from the control group; ^^^, differences from a similar values on the intact side in subjects from the same group.

## Data Availability

Any additional information or data can be requested from the authors.
